# Increase in tuberculosis notification rates among newly arriving male Ukrainian refugees to Norway, 2022 to 2024

**DOI:** 10.2807/1560-7917.ES.2025.30.34.2500633

**Published:** 2025-08-28

**Authors:** Karine Nordstrand, Jacob Dag Berild

**Affiliations:** 1 Norwegian Institute of Public Health, Oslo, Norway

**Keywords:** Tuberculosis, surveillance, migration

## Abstract

The war in Ukraine has caused large population displacements. We report increasing tuberculosis (TB) notification rates among Ukrainian refugees to Norway detected through systematic screening upon arrival and driven by rates among adult males. From 2022 to 2024, there were 73 TB notifications in Ukrainians; incidence among Ukrainian men reached 248 per 100,000 in 2024. In 22 cases, isolated *Mycobacterium tuberculosis* were multidrug-resistant. Adequate TB surveillance and control strategies are important to prevent TB outbreaks, including multidrug-resistant TB, in Europe.

European countries currently host around 5.1 million Ukrainian refugees [1]. Norway is one of few European countries requiring mandatory universal screening for tuberculosis (TB) among asylum seekers and refugees, regardless of their country of origin. We aimed to investigate changes in TB notification rates among newly arrived refugees from Ukraine to Norway by combining aggregate numbers from the Norwegian Directorate of Immigration (UDI) and TB surveillance data from the Norwegian Surveillance System for Communicable Diseases (MSIS).

## Ukrainian refugees in Norway and Europe

By the end of 2024, 91,638 Ukrainians had sought protection in Norway following Russia’s invasion in February 2022. Of these, 42% were adult females and 27% were adult males (Table 1).

**Table 1:** Ukrainian refugees stratified by year of arrival^a^, Norway, 2022–2024 (n = 91,638)

Year	Adult females	%	Adult males	%	Children^b^	%	Total
2022	16,820	47	7,823	22	11,281	31	35,924
2023	14,512	40	11,107	30	10,873	30	36,492
2024	7,203	37	5,790	30	6,229	32	19,222
**Total**	**38,535**	**42**	**24,720**	**27**	**28,383**	**31**	**91,638**

^a^ Figures are based on annual number of applications for protection.

^b^ Younger than 18 years.

Data source: Norwegian Directorate of Immigration.

Among Ukrainian beneficiaries of temporary protection throughout Europe, adult males represented 24% in March 2025, while 45% were adult females [1]. The proportion of adult males among newly arrived refugees to Norway increased from 22% in 2022 to 30% in 2024. The share of adult males among Ukrainian refugees has also increased in Europe.

## Tuberculosis screening of refugees and asylum seekers in Norway

Foreign-born individuals account for around 90% of TB cases in Norway, with more than one third diagnosed within 1 year of arrival [2]. Norwegian legislation stipulates that all refugees and asylum seekers be screened for TB within 14 days of arrival [3]. Screening of Ukrainian refugees entails chest radiography for those 10 years or older, while a questionnaire is used for children younger than 10 years [4].

Tuberculosis is a notifiable disease in Norway, and all cases must be reported to MSIS, managed by the Norwegian Institute of Public Health. The sensitivity of the TB surveillance system is assumed to be high, as notifications come from multiple sources and are routinely checked against TB drug prescriptions. The notification form includes information on country of birth, date of diagnosis, time of arrival to Norway and indication for TB examination (including screening upon arrival). Data completion is high, exceeding 90% for most variables.

## Tuberculosis notifications among newly arrived Ukrainian refugees to Norway

To determine the number of TB cases among newly arrived refugees from Ukraine, we started by identifying all TB cases among people born in Ukraine notified since 2022. We excluded all cases not identified through routine screening upon arrival and all cases already receiving treatment before arrival in Norway. Because screening capacity was at times overwhelmed by the large number of arrivals in the first months after the start of the war, we included all TB cases notified within 1 year of arrival, provided they were reported as identified through routine screening upon arrival. Thus, 73 cases were included, all arriving in Norway between 2022 and 2024 (Figure).

**Figure:**
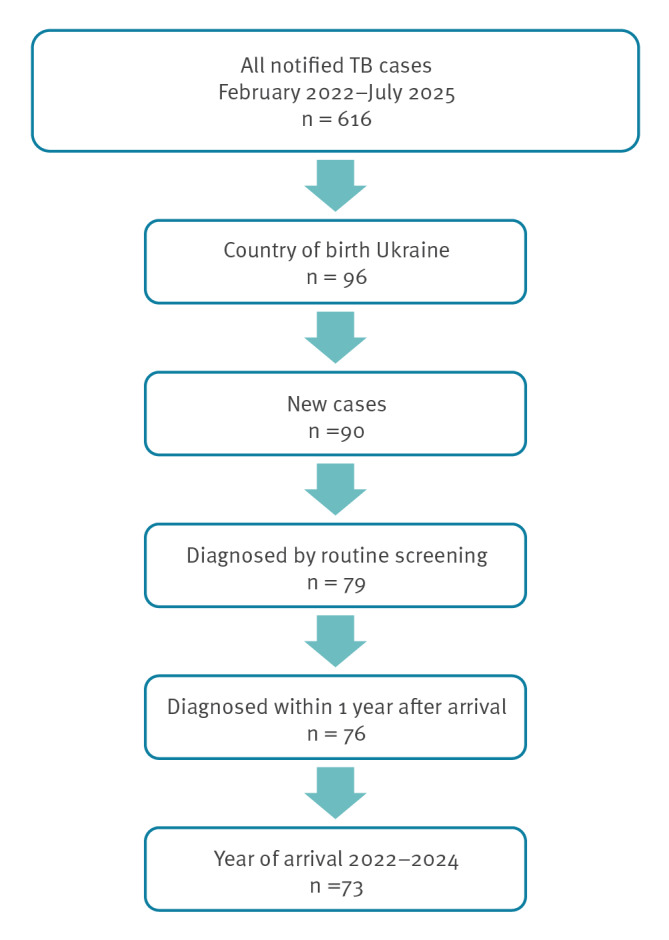
Notified tuberculosis cases among newly arrived refugees from Ukraine, Norway 2022–2024 (n = 73)

TB: tuberculosis.

Data source: Norwegian Surveillance System for Communicable Diseases (MSIS).

Almost all Ukrainians diagnosed with TB in Norway were adults. Since 2022, no Ukrainian-born children younger than 10 years have been notified with TB in Norway. Of the 21 cases notified in 2022, two thirds were adult females and one third were adult males. By 2024, one quarter were adult females and almost three quarters were adult males (Table 2).

**Table 2:** Notified tuberculosis cases among newly arrived Ukrainian refugees, stratified by age group, year of arrival and sex, Norway, 2022–2024 (n = 73)

Age (years)	2022			2023			2024		
	Male	Female	Total	Male	Female	Total	Male	Female	Total
< 18	0	0	0	0	0	0	0	1	1
18–39	3	6	9	8	8	16	5	3	8
40–59	2	6	8	9	4	13	4	2	6
≥ 60	1	3	4	3	0	3	5	0	5
**Total**	**6**	**15**	**21**	**20**	**12**	**32**	**14**	**6**	**20**

Data source: Norwegian Surveillance System for Communicable Diseases (MSIS).

Assuming complete coverage of the arrival screening, we calculated notification rates per year of arrival using notified cases as the numerator and registered refugees as the denominator. We also calculated rate ratios across years, using 2022 as the reference year. We observed an increasing trend in TB notification rates among newly arrived Ukrainian refugees between 2022 and 2024, driven by an increase among adult males from 76.7 per 100,000 in 2022 to 241.8 per 100,000 in 2024 (rate ratio = 3.2; 95% confidence interval: 1.2–8.2) (Table 3).

**Table 3:** Notification rates of tuberculosis disease per 100,000 among newly arrived Ukrainian refugees and stratified by year of arrival and sex, Norway, 2022–2024 (n = 73)

Year of arrival	TB cases	Ukrainian refugees	Notification rate per 100,000 (95% CI)	Rate ratio^a^ (95% CI)
Overall				
2022	21	35,924	58.5 (33.5–83.5)	Reference
2023	32	36,492	87.8 (57.3–118.1)	1.5 (0.9–2.6)
2024	20	19,222	104.0 (58.4–149.6)	1.8 (1.0–3.3)
Adult females				
2022	15	16,820	89.2 (44.0–134.3)	Reference
2023	12	14,512	82.7 (35.9–129.5)	0.3 (0.4–2.0)
2024	5	7,203	69.4 (8.6–130.3)	0.8 (0.3–2.1)
Adult males				
2022	6	7,823	76.7 (15.3–138.1)	Reference
2023	20	11,107	180.1 (101.1–259.0)	2.4 (0.9–5.8)
2024	14	5,790	241.8 (115.1–368.5)	3.2 (1.2–8.2)

CI: confidence interval; TB: tuberculosis.

^a^ Vs 2022 rate.

Data sources: Norwegian Surveillance System for Communicable Diseases (MSIS) and Norwegian Directorate of Immigration.

Twenty-two of the 73 cases had multidrug-resistant TB (MDR-TB), including seven cases of pre-extensively drug-resistant TB. Whole genome sequencing did not indicate transmission between the MDR-TB cases diagnosed in Norway.

## Discussion

Our study shows a notable increase in TB notification rates specifically among Ukrainian adult males and high rates of MDR-TB. The number needed to screen (NNS) to find one TB case in the study population was 1,255 across the 3 years. This includes children younger than 10 years who are primarily screened through a questionnaire. If only adults are considered, 879 people needed to be screened by chest radiography to find one case of TB. In a French survey in 2022 on TB cases detected through active screening among Ukrainian refugees, the NNS was comparable, at 862 [5]. In a similar survey on TB screening of Ukrainian refugees in Germany, also from 2022, the NNS was 545 among those older than 15 years [6]. The rate of MDR-TB was similar to ours in the study from France (27%), but higher (42%) in the study from Germany.

As there is no register for TB screening upon arrival, UDI data on applications for protection served as a proxy for number of screened individuals, assuming all Ukrainian refugees were screened according to national TB legislation. This assumption may not hold true, even though screening coverage has been estimated at above 90% among refugees from Ukraine [7]. Hence, the actual TB incidence among newly arrived Ukrainian refugees to Norway may be higher than our estimates. Furthermore, because of the high number of arrivals directly following the Russian invasion, there may have been more missed cases among refugees arriving in 2022 compared with 2024. However, MSIS data on all TB notifications among Ukrainian adult male refugees, regardless of time of residency and indication for TB examination, show only three additional cases; one with year of arrival 2022 and two with year of arrival 2023. This indicates high sensitivity of the screening system.

Whether the observed increase in TB notification rates in our study is due to higher transmission rates in Ukraine as a consequence of the ongoing war or related to differences in the characteristics of the refugees over time (e.g. age distribution, socioeconomic status), remains uncertain.

Before the invasion, Ukraine had one of the highest TB burdens among the 53 countries in the World Health Organization (WHO) European Region, including MDR-TB [8]. War and conflict impact the epidemiology of infectious diseases by disrupting healthcare systems, causing population displacement and creating conditions that favour disease transmission [9]. According to estimates from the WHO, the TB incidence rate in Ukraine increased from 85 per 100,000 population in 2021 to 112 per 100,000 in 2023 [8], surpassing the commonly used threshold for high TB incidence at 100 per 100,000 population [10]. 

A recent analysis of the impact of the invasion on TB incidence in Ukraine indicates a changing pattern of TB incidence throughout Ukraine during the conflict, with lower rates in conflict-affected areas and higher rates in regions hosting internally displaced people [11]. That study, together with our data, underscores the importance of strengthening TB surveillance and control strategies in areas experiencing population influx.

Screening policies for TB infection and/or disease among migrants vary widely across Europe [12]. In the 2022 Public Health guidance from the European Centre for Disease Prevention and Control, systematic screening for TB disease is only recommended for certain groups of Ukrainian refugees, including people living with HIV, household contacts and other close contacts of individuals with TB disease [10]. According to a 2023 survey among 34 European countries, 13 countries advised screening all refugees from Ukraine for TB, nine countries screened specific groups only, while 12 countries did not screen at all [13].

## Conclusion

In 2024, the TB notification rate among newly arrived Ukrainian refugees to Norway increased to 104 per 100,000 population. While the absolute number of TB cases among Ukrainian refugees to Norway remains low, important differences are observed between different population groups. Screening recommendations and strategies should be adapted to target those most at risk, to enable optimal use of available resources and ensure patients receive timely and necessary care. The evolving epidemiological pattern described above may be of interest to inform policy and interventions directed towards TB prevention and care in European countries receiving refugees from Ukraine. 

## Data Availability

Aggregate TB surveillance data are publicly available, while more granular data are available from the Norwegian Institute of Public Health upon request. Aggregate data on immigration to Norway is publicly available from the Norwegian Directorate of Immigration.
